# Dihydrotachysterol intoxication treated with pamidronate: a case report

**DOI:** 10.1186/1757-1626-3-78

**Published:** 2010-03-26

**Authors:** Mojca Jensterle, Marija Pfeifer, Matjaz Sever, Tomaz Kocjan

**Affiliations:** 1 Department of Endocrinology, Diabetes and Metabolic Diseases, University Medical Centre, Zaloska 7, SI-1000 Ljubljana, Slovenia

## Abstract

**Introduction:**

Hypoparathyroidism is a chronic condition which requires a lifelong substitution with vitamin D analogues and careful monitoring. This is especially true for older patients and older compounds as dihydrotachysterol with longer half-life that might lead to long-lasting hypercalcemic episodes.

**Case presentation:**

A 74-year old male patient with postsurgical hypoparathyroidism who has been successfully supplemented with dihydrotachysterol (1.7 ml/day) for over 50 years presented with neuropsychiatric disturbances, constipation, renal insufficiency and polyuria. Laboratory investigation demonstrated serum calcium 3.7 mmol/L, serum creatinine 180 micromol/L, urine calcium excretion 1.1 mmol/mmol of the creatinine, normal 25 OH vitamin D_3 _and low parathormone and 1,25 di OH vitamin D_3_. Careful history revealed that he has been erroneously taking 2.5 ml of dihydrotachysterol per day for at least 6 to 8 weeks that caused vitamin D intoxication and symptomatic hypercalcemia. He was treated with intravenous saline infusion, prednisolone and 60 mg of intravenous sodium pamidronate. On the fourth day after admission serum calcium dropped rapidly within the reference range. The treatment for hypoparathyroidism had to be reinstituted 10 days after dihydrotachysterol had been discontinued when the patient was switched to shorter acting calcitriol.

**Conclusions:**

Here we reported that the immediate use of pamidronate in addition to classic treatment of dihydrotachysterol intoxication with intravenous saline, diuretics and glucocorticoids is an effective treatment choice that leads to rapid resolution of hypercalcemia.

## Background

Vitamin D intoxication is a rare cause of hypercalcemia. However, it should be considered even after uneventful chronic therapy of hypoparathyroidism, especially in older patients. Common treatment regimens for hypoparathyroidism include colecalciferol (vitamin D_3_) or ergocalciferol (vitamin D_2)_, alfacalcidol (1 alpha-hydroxycolecalciferol) and calcitriol. Colecalciferol and ergocalciferol are the least expensive preparations but have the longest duration of action. This is due to the storage of these compounds in body fat and consequent slow release which may result in prolonged toxicity. The newer preparations, alfacalcidol and calcitriol, which do not require renal 1 alpha hydroxylation, are much more potent, have the advantage of shorter half-life and thereby minimal risk of prolonged toxicity. Calcitriol is the natural active metabolite and, unlike alfacalcidol, does not require the hepatic 25-hydroxylation [1]. Nowadays, it is the drug of choice for treating hypoparathyroidism.

Dihydrotachysterol is only seldom used in the treatment of hypoparathyroidism. It is a half synthetic analog of vitamin D and is converted to the active form in the body. Dihydrotachysterol actions and pharmacokinetics resemble those of vitamin D_3 _including the prolonged toxicity. It stimulates calcium and phosphate absorption from the small intestine, promotes mobilization of calcium from bone to blood and promotes renal tubular reabsorption of phosphate. It is stored in liver, fat, skin, muscle, and bone and excreted in feces. Its peak effect on serum calcium concentration is reached in 2-4 weeks and lasts for about 9 weeks which means a prolonged toxicity. It is of note, that dihydrotachysterol is not detected by the 25 OH D_3 _and 1,25 di OH vitamin D_3 _assays [2].

Vitamin D intoxication is a treatable cause of hypercalcemia. Calcitriol-induced hypercalcemia usually lasts only one to two days due to the short biologic half-life of the compound. Discontinuing the calcitriol, increasing salt and fluid intake or additional hydration with intravenous saline may be the only treatment needed. In contrast, hypercalcemia induced by intoxication with longer lasting preparations such as dihydrotachysterol, vitamin D_3 _and vitamin D_2 _takes longer to resolve because of deposition of ingested vitamin D in fat and its consequent slow release. Therefore, more aggressive therapy including intravenous hydration, diuretics and glucocorticoids is needed.

However, it has been demonstrated that the major cause of hypercalcemia due to vitamin D intoxication is actually the increased bone resorption [3]. If this is the case, specific inhibitors of bone resorption as bisphosphonates might provide more effective treatment than conventional therapy. We carried out a systematic review of most previously published case reports using bisphosphonate treatment in patients with vitamin D intoxication. The results are presented in Table 1[4-15]. We also found three review articles that are reported in the following paragraph.

**Table 1:** Systematic review of previously published case reports using bisphosphonate treatment in patients with vitamin D intoxication.

Ref.	Age, Sex	Underlying disease	Vitamin D compound	Serum Ca/P (mg/dl)	Creatinine (mg/dl)	PTH (pg/ml)	25 OH D3 (ng/ml)	Treatment	Other treatment	Response time
4.	40 years, M	Hypoparathyroidism	Ergocalciferol 100.000 U/day for 3 years	13.0	4.2		121	Disodium etidronate 400 mg	Hydration, prednisone	42 weeks
5.	7 months	None	Vitamin D					Alendronate		
6.	3 months, M	None	Vitamin D 1.200.000 U/day	18.5/3.2		< 1.0	360	Alendronate 5-10 mg/day	Hydration, diuretics	18 days
7.	31 years, F	Hypoparathyroidism	Dyhydrotahysterol 4 mg/day for 6 months	4.1 mmol	5.5			Pamidronate 20 mg once	Hydration, diuretics	20 weeks
8.	16 months, M	None	Vitamin D	18				Pamidronate	Hydration, furosemide, glucocortioids	
9.	8 months, F	Vitamin D deficiency	25-hydroxy vitamin D 1.200.000 U	14.2/4.8		< 3	210	Alendronate 5 mg/day	Hydration, furosemide	3 days
9.	35 days, M	Vitamin D deficiency	25-hydroxy vitamin D 600.000 U	14.5/5.9		< 3	240	Alendronate 5-10 mg/day	Hydration, furosemide	8 days
10.	3 months, M	None	Vitamin D 2.560.000 U	16.4/6.7			268	Pamidronate 20 mg twice	Hydration, furosemide, prednisolone	36 hours
11.	6 months	None	Vitamin D					Pamidronate 1 mg/kg/day twice	Hydration, furosemide, calcitonine, prednisolone	2 days
12.	11 months, F	None	Cholecalciferol > 900.000 U	18		1.84	200	Alendronat 5 mg/day for 21 days	Calcitonine, glucocorticoid, oral phosphates	3 weeks
12.	4 months, F	Misdiagnosed for rickets	Cholecalciferol 600.000 U	14.9		4.1	> 160	Pamidronate, 1 mg/kg/day for 9 days, alendronate 10 mg/day for 6 weeks	Hydration, glucocrticoids, calcitonin, phosphates	9 weeks
13.	77 years, F	Osteoporosis	Vitamin D 50.000 U/day for 6 days	21	1.9		280	Pamidronate 90 mg once	Hydration	24 hours
14.	7 years, M	Vitamin D deficiency	Vitamin D 300.000 U/day for 15 days	12.1/3.4			> 400	Alendronate 5-10 mg	Hydration, diuretic	16 days
15.	76 years, F	Hypoparathyroidism	Ergocalciferol 100.000 U/day, 12 years	15.3/3.0				Pamidronate 45 mg five times	Hydration, prednisolone	11 months

Selby et al. [3] observed a rapid reduction in plasma calcium concentration with pamidronate in a group of patients with vitamin D intoxication. In the same group corticosteroids had a more delayed effect. They concluded that hypercalcemia of vitamin D intoxication is mediated by increased bone resorption and bisphosphonates should have a role in its management. Rizzoli et al. [16] reported that intravenous administration of clodronate corrected hypercalcemia and hypercalciuria of vitamin D intoxication whereas prednisolone therapy barely affected biochemical values. Finally, Quack et al. [17] reported successful application of single dose pamidronate 15 mg even in four patients with dihydrotacysterol intoxication regardless of their renal failure.

To our knowledge, the combination of conventional treatment and pamidronate, has never been introduced simultaneously and immediately after admittance in a patient with dihydrotachysterol intoxication. Considering the extremely brisk response in our patient we believe that this is an effective and advantageous treatment regimen which also enables rapid reinstitution of the substitution therapy.

## Case presentation

A 74-year old male patient was sent to the emergency neurologic outpatient clinic because of progressive confusion, lethargy, dysarthria, fatigue and weakness in the last week. He complained of constipation, anorexia and vague abdominal pain. The CT scan of the brain that was immediately performed by the neurologist was normal.

An interview with his son who arrived a few hours later revealed that the patient had been treated for hypertension and postsurgical hypoparathyroidism without any other important comorbidity. He was followed regularly at an endocrinology outpatient clinic once yearly. His last visit two months before current admission showed normal laboratory values including serum calcium, creatinine and phosphate. He had near-total thyroidectomy for benign goiter back in 1955. Following the surgery he had episodes of tetany and postsurgical hypoparathyroidism was diagnosed 2 years later. A therapy with dihydrotachysterol was introduced in 1957. In 1972 he had a single episode of renal colic which ended with spontaneous stone passage. In 1995 thiazide diuretic was added to the dihydrotachysterol due to hypercalciuria. There were several attempts to introduce shorter acting vitamin D derivatives during 50 years of the disease history, but finally the patient had always rejected all new medications and switched back to dihydrotachysterol.

On examination, he was lethargic, dysarthric with slow tongue movements and poor cognitive estimates, hypertensive (blood pressure was 165/95 mmHg), dehydrated. Cardiovascular, respiratory and abdominal examinations were unremarkable. Cranial nerve examination was normal. He had bradykinesia with decreased power of the limbs, in association with decreased reflexes and slightly increased tone in the axial parts of the limbs. Plantar responses were normal. After admission we noted also severe constipation and polyuria few days later.

When laboratory investigations demonstrated serum calcium of 3.7 mmol/L, serum creatinine 188 μmol/L and urea 16.2 mmol/L, he was admitted to the endocrinology department where some additional tests were done. Urinary calcium excretion in the 24 hours collection was increased to 1.1 mmol/mmol of creatinine. Alkaline phosphatase, phosphate, 25 OH vitamin D_3_, ACE (angiotensin converting enzyme), PSA and CEA were normal. Serum parathormone and 1,25 di OH vitamin D_3 _were low (Table 2). The QTc interval on ECG was within reference range. Test of occult blood in stool specimen was negative. Abdominal ultrasound showed normally sized kidneys, no renal calculi and no nephrocalcinosis. Chest X-ray was normal.

**Table 2:** Laboratory findings upon admission.

	Patient	Reference range
S-calcium	3.7	2.1-2.6 mmol/L
S-phosphate	1.1	0.8-1.4 mmol/L
S-creatinine	188	44-97 μmol/L
Urinary calcium excretion in the 24 hour collection	1.1	0.13-0.45 mmoL/mmoL of the creatinine
Alkaline phosphatase	1.08	< 1.5 μcat/L
Parathormone	< 3	12-72 ng/L
25 OH D3	46	> 75 nmol/L
1,25 di OH vitamin D_3_	14	40-155 pmol/L
PSA	1.36	< 3 μg/L
ACE	0.17	0.13 - 0.47 μcat/L

**Figure 1:**
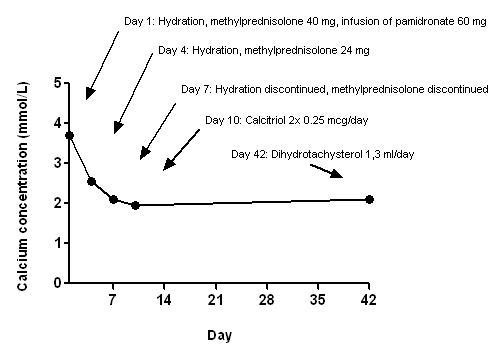
**Laboratory findings and treatment follow up**. Reference range for serum calcium is 2.1-2.6 mmol/L.

Based on the history the patient was diagnosed with vitamin D intoxication due to dihydrotachysterol overdose. Differential diagnosis included malignancy and sarcoidosis. However, according to the data collected they seemed unlikely and therapy was initiated before their proper exclusion. The patient was treated according to conventional treatment regimen for this condition with intravenous saline infusion, careful administration of loop diuretic (furosemide) and methylprednisolone. We additionally applied 60 mg of pamidronate sodium in slow intravenous infusion over 4 hours. Two days after the admission the patient became less confused. His communication improved and he admitted that he had been mistakenly taking 75 drops (2.5 ml) of dihydrotachysterol per day for the last 6 to 8 weeks instead of his usual daily dose of 50 drops (1.7 ml). The reason for this sudden change of his regimen remained a mystery. On the fourth day after admission serum calcium dropped rapidly to the reference range. The observed constipation and polyuria resolved in few days after normalization of serum calcium concentrations. The treatment for hypoparathyroidism had to be reinstituted 10 days after dihydrotachysterol had been discontinued. The patient was now easily switched to shorter acting vitamin D derivate calcitriol (0.25 μg twice per day) which maintained a normal serum and urinary calcium concentration without a thiazide. However, two weeks after the discharge from the department of endocrinology he discontinued calcitriol and again started taking dihydrotachysterol, since old habits die hard. Laboratory values and treatment during follow up are reported in Figure 1.

## Conclusion

Most symptoms of hypercalcemia are non-specific and are difficult to be perceived by physicians. It should be realized that vitamin D in all its forms can be a demanding drug to use in a lifelong treatment of hypoparathyroidism. Continuous vigilance is required, especially in older people. In comparison to short acting vitamin D derivatives hypercalcemic episodes with dihydrotachysterol appear to last longer and may occur with higher incidence. Whenever possible, a switch to shorter acting vitamin D derivatives is recommended. The first line treatment of vitamin D intoxication includes intravenous hydration, diuretics and glucocorticoids. Since hypercalcemia of hypervitaminosis D is predominantly caused by increased bone resorption, bisphosphonates might provide more effective treatment. We reported that the instant use of pamidronate and methylprednisolone for moderate to severe hypercalcemia due to vitamin D intoxication with dihydrotachysterol is an effective treatment choice. Considering the prompt response we believe that bisphosphonates should be initiated in the early stages of treatment of vitamin D intoxication.

## Consent

Written informed consent was obtained from the patient for publication of this case report and accompanying tables. A copy of the written consent is available for review by the Editor-in-Chief of this journal.

## Competing interests

The authors declare that they have no competing interests.

## Authors' contributions

MJ was a major contributor in writing the manuscript. MP has been involved in revising the manuscript critically for important intellectual content. MS contributed in writing the manuscript. TK has made substantial contributions to conception and design of the treatment regimen. All authors read and approved the final manuscript.
